# P-1556. A Retrospective Evaluation of Methenamine Safety and Efficacy in An Older Population at an Academic Medical Center

**DOI:** 10.1093/ofid/ofae631.1723

**Published:** 2025-01-29

**Authors:** Jysheng Hou, Rodrigo M Burgos, Larry H Danziger

**Affiliations:** University of Illinois at Chicago, College of Pharmacy, Chicago, Illinois; University of Illinois at Chicago, Chicago, Illinois; University of Illinois at Chicago, Chicago, Illinois

## Abstract

**Background:**

Urinary tract infections represent a significant cause of morbidity and mortality in older adults. Limited methods exist to prevent recurrent UTI (rUTI) in this high-risk population. Prophylactic low-dose antibiotics are often considered for this purpose, but can be associated with increased adverse effects and development of resistant pathogens. The urinary antiseptic agent methenamine has re-emerged as an antibiotic-sparing alternative for prevention of rUTI, however its role in therapy for older adults is not well characterized.

**Figure:**
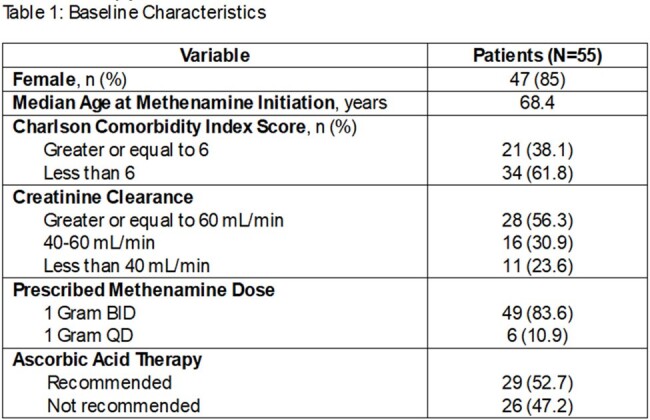

**Methods:**

Retrospective chart review identified patients prescribed methenamine from 2017 to 2023. Excluded were patients on prophylaxis aged < 60 years at the time of initiation, and those with < 3 months duration. Patients who received antibiotics for breakthrough UTI while on methenamine were included. Patient histories were reviewed 1 year prior to methenamine initiation through the end of prophylaxis. Collected were patient demographics, UTI history prior to methenamine, time to recurrence, laboratory values and adverse effects.

**Figure:**
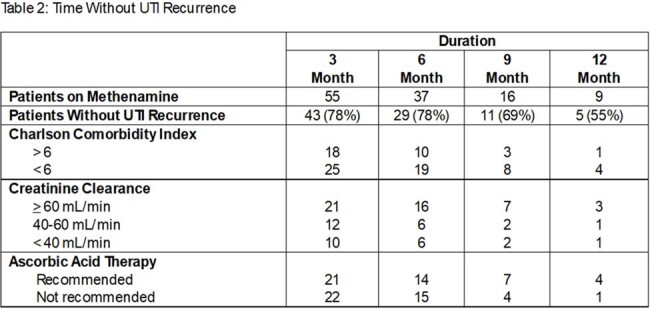

**Results:**

247 patients were prescribed methenamine during the study period. 55 patients met inclusion criteria. On average, methenamine duration was 8.75 months. 27 patients had creatinine clearance (CLcr) values < 60 mL/min. 29 patients received a concurrent recommendation for ascorbic acid. Use of methenamine provided ≥ 3 months without UTI recurrence in 43 patients (78%) with a wide range of Charlson comorbidity index scores and CLcr values, and 29 patients did not have documented UTI for the duration of methenamine therapy. Seven reports of adverse effects were documented, 3 of which led to discontinuation of therapy.

**Figure:**
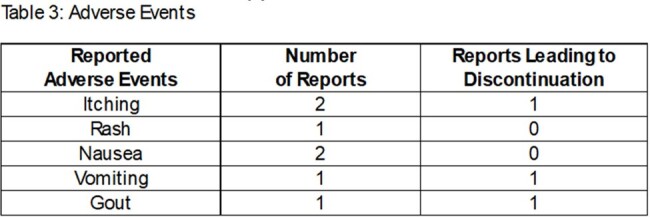

**Conclusion:**

In a population ≥60 years of age, methenamine appears to be safe and effective at preventing recurrent UTIs. The number of patients without UTI decreased with time on methenamine, however 55% of patients who remained on prophylaxis at one year had no documented recurrence. Concurrent ascorbic acid therapy was not consistently recommended in this study population, and was not associated with increased methenamine efficacy. Lower CLcr did not appear correlated with lower efficacy or increased adverse effects of methenamine.

**Figure:**
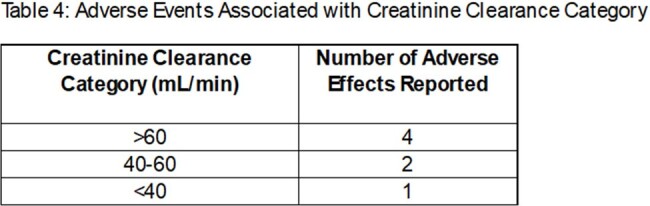

**Disclosures:**

**All Authors**: No reported disclosures

